# The Importance of Early Treatment in Nasal Obstructions

**Published:** 1894-08

**Authors:** Robt. E. Moss


For Texas Medical Journal.

### THE IMPORTANCE OF EARLY TREATMENT IN NASAL OBSTRUCTIONS.

BY ROBT. E. MOSS, M. D.

[Read before the Western Texas Medical Society, June 28, 1894.]

IT WOULD almost seem like threshing over straw to write upon this hackneyed subject, but the great number of neglected cases and the careless indifference on the part of the majority of general practitioners, is my excuse for this paper to-night.

If we study the anatomical construction and physiological function of the nose and accessory cavities, we will be impressed with the fact that it has an important part to play in giving physical beauty, health and happiness to each individual.

The nose, as we know, has three turbinated bones, scroll shaped, on either side, which are covered with mucous membrane, studded with glands, and richly supplied with blood vessels. There are openings from the antrum of Highmore, ethmoid, sphenoid and frontal sinuses, and the lachrymal ducts. Diseased conditions of the nose readily extend to all of these, either by continuity of tissue, or from destruction. The chief function of the nose, of course, is respiration; but the importance of nasal, over mouth breathing, is not properly understood, generally speaking, and if understood, but slightly regarded. The nose, with its great surface of moist mucous membrane, catches the dust, warms and moistens the air before it reaches the delicate structure of the air vesicles. This is a matter of the greatest importance, for who among us has not seen the marked change in health, intelligence, personal appearance and rapid growth of the dull, listless, mouth-breathing child who has been relieved by operation? A vast number of these children will breathe through the nose during the day, but you will find that they sleep with the mouth open at night, and you will invariably find they are extremely susceptible to atmospheric changes and various bodily ills. The anxious mother will tell you her child takes cold easily and complains a great deal, in fact is never well, and she doesn't know what to do, etc., etc.

It matters but little whether this obstruction of the nose is due to polyps, hypertrophy, or indirectly by chronic enlargement of pharyngeal and faucial tonsils, the effect is the same. There may be, and generally is, an underlying constitutional diathesis which predisposes to this condition; yet constitutional treatment, no matter how skillfully directed, will not relieve these little sufferers. There can be no question that taking in the air without its proper amount of moisture or heat, as is done in all mouth breathing, the delicate structures in the lungs are affected, the blood imperfectly oxygenated, the system badly nourished, and a general predisposition to acquire almost any disease.

In regard to the causation of these hypertrophies, generally speaking, we may group them together; for the conditions giving rise to one form, as a rule, will produce them all. It is true, we may have them as separate and distinct lesions, yet in many respects they are similar. We find, however, where the glandular structures are involved to a greater extent, that there is doubtless a constitutional dyscrasy—such as inherited syphilis or tuberculosis—which shows its predilection for lymphatic and glandular structures. Repeated coryzas due to improper care in dress, sleeping apartments, atmospheric conditions, and a general lack of proper hygienic management, leads to deep-seated inflammations. As a result, we have hypertrophy, interstitial deposits, and a general hyperplasia, which defies the best directed treatment.

Mothers must be taught that a common cold is not the insignificant thing our predecessors in medicine led them to believe. It is the duty of the family physician to instruct them, in a general way, and when his attention is called to the fact that the child has a discharge from the nose, and can't breathe well at night, he should not dismiss the subject with a shrug of the shoulders or by saying it will be well in a short time. There are thousands of suppurative ear troubles due to neglect on the part of the attendant in ignoring a coryza. The inaccessibility of the naso-pharyngeal space in children is probably an excuse for not examining it more frequently, and yet it is an easy matter to insert the index finger behind the palate and carefully examine the parts.

The eruptive fevers are responsible for, or causative of a great number of these cases, especially measles. The patient's nose positively receives little, if any, treatment in attacks of measles, yet coryzas are very common, and suppurative troubles of the middle ear and mastoid antrum far from uncommon. Scarlet fever and diphtheria affect the nose very frequently, causing mouth breathing, with its baneful effects upon a system whose vitality is already at a low ebb. Then there are scores of suppurative ear cases complicating these diseases, and the cause will frequently be found due to nasal obstruction. Mothers should be taught to watch their children in regard to mouth breathing, snuffles, or a discharge from the nose, at any time, and especially following any of the exanthemata, or other febrile diseases. It is in the incipiency of attacks that treatment is of any avail. If we wait until true hypertrophy has become developed, it then becomes necessary to resort to surgical methods to remove obstructions, relieve pressure, and open the nasal cavities sufficiently to relieve the patient altogether of mouth breathing.

As to the treatment in the early stages of nasal obstructions where there is acute coryza from any cause, it is very simple. Cleanliness is the main thing; using any alkaline spray, such as a solution of Seiler's tablets, Dobell's solution, or listerine and boric acid, or any combination the individual practitioner may prefer. The main point is the spray should be used quite warm, and frequent enough to clear the nose of all tenacious plugs of mucus. The diet, dress, baths, general tonics, exercise, and the two greatest of all restoratives, sunshine and fresh air, should receive due attention, and the mother be given positive directions. It is too often the case that after the prescription has been written, the physician will give a few general directions and the mother be left in doubt how to act, except upon her own judgment or that of some neighbor who prides herself upon knowing more about treating children than the doctors.

After true hypertrophy of tissue has occurred we find sprays as a rule, very little, if any benefit. In slight hypertrophies of the turbinated tissues cauterizing will usually answer, and we generally prefer chromic acid fused on the end of a slender probe carefully applied after the parts have been well cocainized. Where the hypertrophy is *very* marked, the Bosworth saw, scissors or snare will be required, and here I would suggest that good judgment and skill are needed. I will venture the assertion that no part of the body has suffered more bad surgery either by omission or commission than the nose. It is only a few years ago that we knew anything about how or what to do in many of the conditions that we now treat intelligently and with as much success as similar diseased processes in other parts of the body. The gynecologists like to twit the "skinologists" about not being able to cure catarrh, and yet, how many of them ever cure a case of true hypertrophy or hyperplasia of the uterus? It is in cases of recent subinvolution that the results are highly satisfactory when proper treatment has been skillfully applied; just so in nasal obstruction due to congestion, and not hypertrophy.

There are many abnormalities, as tumors, polyps, exostoses, fractured and deviated septums, etc., but these all require special skill as a rule, to remove or properly treat them. I will detail a case or two to show the results of treatment in different forms of obstruction.

Miss S., aet 24, home, Michigan, sent here by family physician, thinking she had incipient phthisis, She was referred to me by her attending physician in the city on account of a chronic pharyngitis sicca. I found a long ridge of bone upon the septum on left side almost completely occluding that nostril; the turbinated tissues were hypertrophied, and nasal breathing an impossibility. There was middle ear catarrh and the hearing for watch reduced to 12-40 right and 6-40 left. She had lost flesh, chronic cough, light night sweats, and felt sure she was going to die. I removed the obstructions with saw, treated ears by inflation with catheter and douch apparatus containing menthol, iodine and albolene. In six weeks she had gained twenty pounds and the tinnitus was checked. At the end of three months the cough had ceased, a further gain of five pounds in weight, and the hearing in right ear $\frac{40}{40}$, left $\frac{24}{40}$.

Willie L., aet 1½; was called December 28th, with family physician, on account of suppuration of both ears and acute abscess of mastoid on right side. I found nose obstructed, pharyngeal tonsil filling vault of pharynx, profuse discharge from both external auditory canals, right mastoid very much swollen and fluctuating. He had high fever 104 F., very restless anæmic, yellow skin, refusing his bottle, and the picture of malnutrition. I advised immediate operation for the adenoid tissue and the mastoid at the same sitting. We operated that afternoon, opening the abscess and going as deep as the antrum, finding there some beginning caries which was curetted away, wound thoroughly, cleaned and packed with gauze. I then removed the adenoid tissue, and directed the external canals to be carefully syringed three times a day, as the discharge was profuse, and the child to have a tenth of a grain calomel tablet three times a day. At the end of four weeks discharge had ceased in both ears, mastoid opening almost completely closed, and the child's appearance transformed.

Miss B., aet 12, constant trouble with her throat, slight cough, sleeps with mouth open at night, and propped with pillows almost to sitting posture to sleep at all, and when she takes the slightest cold sleep is almost an impossibility. The turbinated tissues showed true hypertrophy and vault of pharynx literally filled with enlargement of pharyngeal tonsil. Assisted by family physician who gave chloroform, I removed the pharyngeal and both faucial tonsils which were also hypertrophied and follicular. At the end of two weeks she came to my office when I removed the thickened turbinated tissues with saw and snare. Six weeks after first operation she can sleep on back without a pillow and with mouth closed. There is no sore throat, cough has disappeared, appetite regular and not capricious as before, and her appearance would suggest perfect health.

I could detail dozens of such cases from my case book, but these will suffice to show the suffering caused by neglect of treatment and the marked relief obtained by thorough radical measures promptly applied.

In closing I would urge the great importance and benefit of early recognition and proper treatment to prevent alterations in nutrition, thereby predisposing to pulmonary troubles. The lack of attention on the part of the family physician and patient, or parent; and the great success of early treatment. I would also call attention to the sad, dull, listless faces presented in this class of patients and the irreparable damage resulting from neglect of, or improper treatment.

